# Ventricular arrhythmias in mouse models of diabetic kidney disease

**DOI:** 10.1038/s41598-021-99891-9

**Published:** 2021-10-18

**Authors:** Kenneth R. Laurita, Shenaz Khan, Tracy McMahon, Adrienne T. Dennis, Vincent Li, Robert Gaivin, Hima Sapa, Ji-dong Fu, Jeffrey R. Schelling

**Affiliations:** 1 Department of Medicine, Case Western Reserve University, MetroHealth Campus, 2500 MetroHealth Drive, R654, Cleveland, OH 44109 USA; 2 Department of Biomedical Engineering, Case Western Reserve University, Cleveland, OH 44106 USA; 3 Department of Medicine, University Hospitals Cleveland, Case Western Reserve University, Cleveland, OH 44106 USA; 4 Department of Physiology and Biophysics, Case Western Reserve University, Cleveland, OH 44106 USA; 5 Present Address: Department of Physiology and Cell Biology, The Ohio State University, Columbus, OH 43210 USA

**Keywords:** Arrhythmias, Chronic kidney disease

## Abstract

Chronic kidney disease (CKD) affects more than 20 million people in the US, and it is associated with a significantly increased risk of sudden cardiac death (SCD). Despite the significance, the mechanistic relationship between SCD and CKD is not clear and there are few effective therapies. Using optical mapping techniques, we tested the hypothesis that mouse models of progressive diabetic kidney disease (DKD) exhibit enhanced ventricular arrhythmia incidence and underlying arrhythmia substrates. Compared to wild-type mice, both *Lepr*^*db/db*^* eNOS*^*−/−*^ (2KO) and high fat diet plus low dose streptozotocin (HFD + STZ) mouse models of DKD experienced sudden death and greater arrhythmia inducibility, which was more common with isoproterenol than programmed electrical stimulation. 2KO mice demonstrated slowed conduction velocity, prolonged action potential duration (APD), and myocardial fibrosis; both 2KO and HFD + STZ mice exhibited arrhythmias and calcium dysregulation with isoproterenol challenge. Finally, circulating concentrations of the uremic toxin asymmetric dimethylarginine (ADMA) were elevated in 2KO mice. Incubation of human cardiac myocytes with ADMA prolonged APD, as also observed in 2KO mice hearts ex vivo. The present study elucidates an arrhythmia-associated mechanism of sudden death associated with DKD, which may lead to more effective treatments in the vulnerable DKD patient population.

## Introduction

In the US chronic kidney disease (CKD) and diabetes affect more than 20 million^1^, and 30 million people (https://www.diabetes.org/resources/statistics/statistics-about-diabetes), respectively. Diabetic kidney disease (DKD) is overwhelmingly the most common cause of progressive CKD and is associated with a significantly increased risk of cardiovascular disease mortality^2^. Of deaths in end stage renal disease (ESRD) patients, i.e., requiring dialysis or renal transplantation, the proportion attributable to sudden cardiac death (SCD), which is most commonly due to ventricular tachycardia/fibrillation (VT/VF), ranges from 18 to 25%^3–7^. Less severe kidney dysfunction is also associated with significant SCD risk^8,9^, which amplifies the premise that decreased glomerular filtration rate (GFR) is a risk for VT/VF and SCD. With an annual ESRD mortality rate exceeding 20%, SCD is therefore responsible each year for tens of thousands of deaths among CKD and ESRD patients, which is unquestionably a major public health concern.

Although CKD and ESRD are established risks for SCD, most clinical cardiology trials exclude patients with estimated glomerular filtration rate (eGFR) < 30 ml/min/1.73m^2^^10^. It is therefore unclear whether findings from landmark studies are applicable to this vulnerable population. For example, guidelines for implantable cardiac defibrillators (ICDs) to prevent SCD are established in the general population, but recommendations for CKD patients remain unclear. Meta-analyses that have addressed ICD prophylaxis of SCD in CKD patients are conflicting^11,12^, and the only randomized trial to evaluate ICD effectiveness in ESRD demonstrated no benefit^7^. These findings raise important questions about whether the pathophysiology of SCD is unique in the context of CKD, and whether circulating factors, which are not efficiently cleared by diseased kidneys, may be toxic to the heart. Furthermore, despite the identification of numerous plausible mechanisms that may link SCD with CKD^13^ the absence of an effective treatment remains a significant barrier.

An additional impediment to kidney disease-associated arrhythmia research has been the lack of a small animal model of DKD, which exhibit the cardinal features of glomerular and tubulointerstitial pathology, as well as progressive decreases in GFR. Mice with an inactivating point mutation in the leptin receptor (*Lepr*^*db/db*^) are commonly employed as a model of type 2 diabetes. *Lepr*^*db/db*^ mice develop some DKD traits, such as glomerulosclerosis and albuminuria, but not key attributes such as tubular atrophy or GFR decline. Mice with homozygous deletion of endothelial nitric oxide synthase gene (*eNOS*^*−/−*^) develop hypertension, but not diabetes or significant GFR decline^14^. However, *Lepr*^*db/db*^ combined with *eNOS*^*−/−*^ (*Lepr*^*db/db*^* eNOS*^*−/−*^) permits a faithful phenocopy of type 2 diabetes and DKD, including decreased GFR, interstitial fibrosis and tubular atrophy^14,15^. *Lepr*^*db/db*^* eNOS*^*−/−*^ mice also die suddenly and prematurely^14,16–19^, suggesting that the final event may be an arrhythmia and SCD.

Studies in diabetic *Lepr*^*db/db*^ or hypertensive *eNOS*^*−/−*^ mice on a variety of genetic backgrounds suggest susceptibility to VT. Hearts from *Lepr*^*db/db*^ mice had a lower threshold for VT due to repolarization abnormalities, with prolonged action potential and calcium transient durations, as well as slowing of intracellular calcium decay and conduction velocity^20^. VT susceptibility has also been noted in *Lepr*^*db/db*^ mice following ischemia, due to increased intracellular Na^21^. Cardiac electrophysiology studies in *eNOS*^*−/−*^ mice revealed enhanced ventricular ectopy following Na^+^/K^+^-ATPase inhibition with ouabain or digoxin^22,23^, prolonged action potential and isoproterenol-induced early after-depolarizations from enhanced L-type calcium channel currents^24,25^. Despite the validated DKD phenotype in *Lepr*^*db/db*^* eNOS*^*−/−*^ (2KO) mice, electrophysiological and arrhythmia characterization has never been evaluated in this model.

Obesity is a component of type 2 diabetes, and also a risk for VT^26–29^, but there are only a few animal models of obesity-induced arrhythmias. Zucker diabetic fat rats demonstrate conduction velocity slowing, perhaps by decreasing Na currents and a connexin-43-dependent mechanism^30^, and mice fed a high fat diet (HFD) develop increased myocardial lipid content, which can impair repolarization due to a decrease in potassium channel expression, causing ventricular tachycardia and SCD^31^. Neither model displays decreased GFR. Streptozotocin (STZ) has been used to induce type 1 diabetes and DKD in rodents, but the high doses required to completely obliterate pancreatic β-cells, inflict tubular toxicity, which may confound the renal phenotype^32^. However, low dose STZ combined with a HFD second hit mimics obesity, type 2 diabetes and DKD, including decreased GFR^19,32–34^.

We hypothesized that the well-characterized mouse models of DKD, *Lepr*^*db/db*^* eNOS*^*−/−*^ and HFD + low dose STZ, would exhibit enhanced ventricular arrhythmia incidence and arrhythmia substrates. Indeed, both mouse models demonstrated inducible arrhythmias, in response to programmed electrical stimulation and isoproterenol challenge. We also demonstrate the feasibility of identifying circulating factors, which may mediate arrhythmogenicity in DKD.

## Results

**Figure 1:**
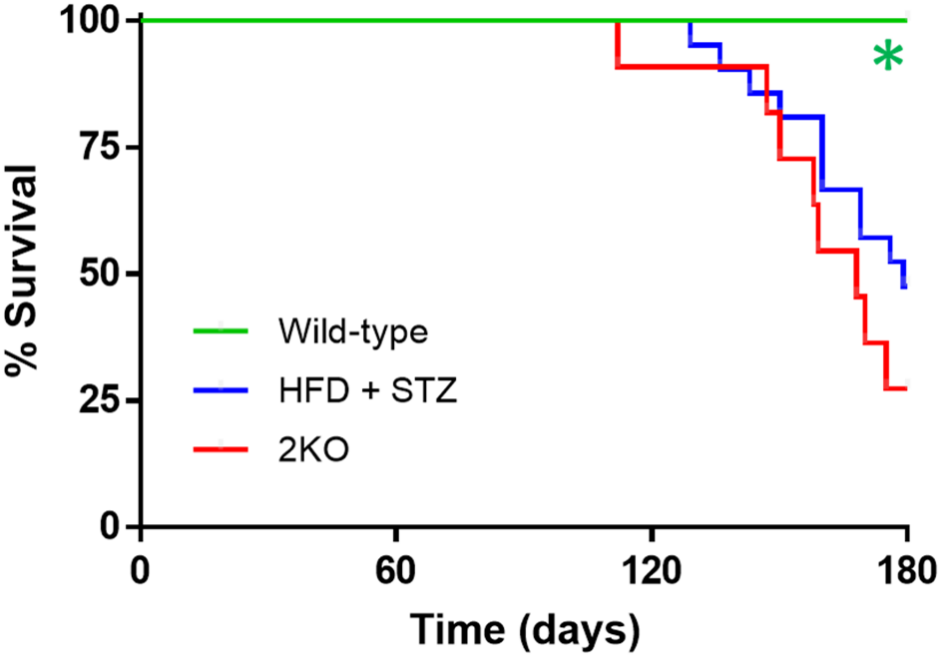
Kaplan–Meier survival curves for in wild-type (N = 8), *Lepr*^*db/db*^* eNOS*^*−/−*^ (2KO, N = 11), and high fat diet plus low dose STZ-treated (HFD + STZ, N = 20) mice. *P < 0.05 by χ^2^ and Mantel-Cox log-rank test compared to other groups.

To establish validity of mouse model susceptibility to VT/VF in the context of DKD, we selected genetic (*Lepr*^*db/db*^* eNOS*^*−/−*^, hereafter referred to as 2KO) and inducible (six months HFD + low dose STZ at three months, hereafter referred to as HFD + STZ) models of type 2 diabetes and DKD for further study. Both models were bred to a congenic C57BLKS/J genetic background, which renders a more severe DKD phenotype. In particular, both strains develop progressive decreases in GFR^19^, which is a risk for VT/VF and SCD in humans^3–9^. Baseline characteristics for wild-type mice and the two DKD strains are shown in Table S1 (supplement). Figure 1 reveals increased mortality of both DKD models compared to wild-type mice. We previously demonstrated that GFR values were reduced by 56% in 2KO and 36% in HFD + STZ mice^19^, which suggests that end stage kidney disease is not the cause of death. Necropsy findings demonstrated no gross abnormalities of any major organs. Although mice had hyperglycemia and significant GFR declines, we were unable to predict imminent death, and we therefore postulated that SCD could be the etiology.

**Figure 2:**
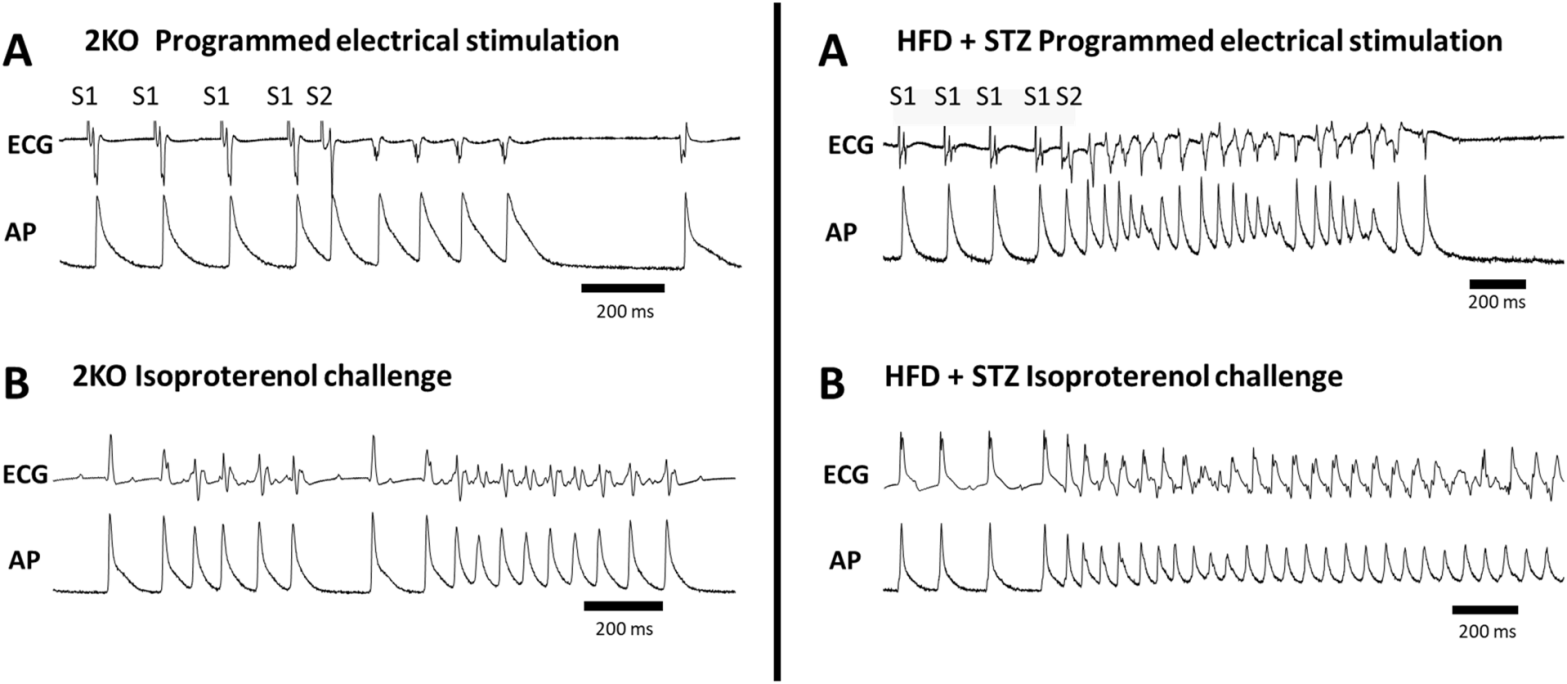
Arrhythmias induced by programmed electrical stimulation **(A)**, and by isoproterenol challenge **(B)** in Lepr ^db/db^ eNOS ^−/−^ (2KO) mice (left) and HFD + STZ mice (right). ECG and action potential (AP) traces were recorded from Langendorff perfused hearts. *AP* action potential, *ECG* electrocardiogram, *S1* baseline pacing, *S2* premature stimulus.

To address the mechanisms of SCD in CKD, optical mapping^35,36^ was performed in Langendorff perfused mouse hearts to assess VT/VF inducibility and to measure electrophysiological abnormalities that can cause arrhythmias (i.e., arrhythmia substrates). Shown in Fig. 2 are examples of arrhythmias induced by programmed electrical stimulation (PES, Panel A) and isoproterenol challenge (Panel B) in 2KO (left) and HFD + STZ mice (right). Summary data (Table 1) show a higher total occurrence of VT/VF in DKD strains compared to wild-type mice, including one instance of spontaneous VT (S2 Video, S1 Video shows normal sinus rhythm for comparison). Arrhythmias occurred more commonly during isoproterenol challenge, which is consistent with abnormal impulse formation (e.g., Ca^2+^-mediated triggered activity), compared to PES, which is consistent with abnormal impulse conduction (e.g., reentry). Furthermore, the incidence of PES and isoproterenol-induced arrhythmia was similar between the two DKD mouse models. These results suggest that both models exhibit a similar arrhythmia phenotype.

**Table 1:** Arrhythmia inducibility in wild-type (C57Bl-KS/J), *Lepr*^*db/db*^* eNOS*^*−/−*^ (2KO) and high fat diet + low dose STZ treated (HFD + STZ) mice.

Mouse strain	Arrhythmia (%)	PES (%)	ISO (%)
Wild-type	2/15 (13%)	1/14 (7%)	1/15 (7%)
2KO	6/11 (55%)*	2/9 (22%)	4/10 (40%)
HFD + STZ	8/13 (62%)*	3/12 (25%)	6/13 (46%)*

*PES* programmed electrical stimulation, *ISO* isoproterenol infusion.

*P < 0.05 compared to wild-type by Fisher’s exact test.

**Figure 3:**
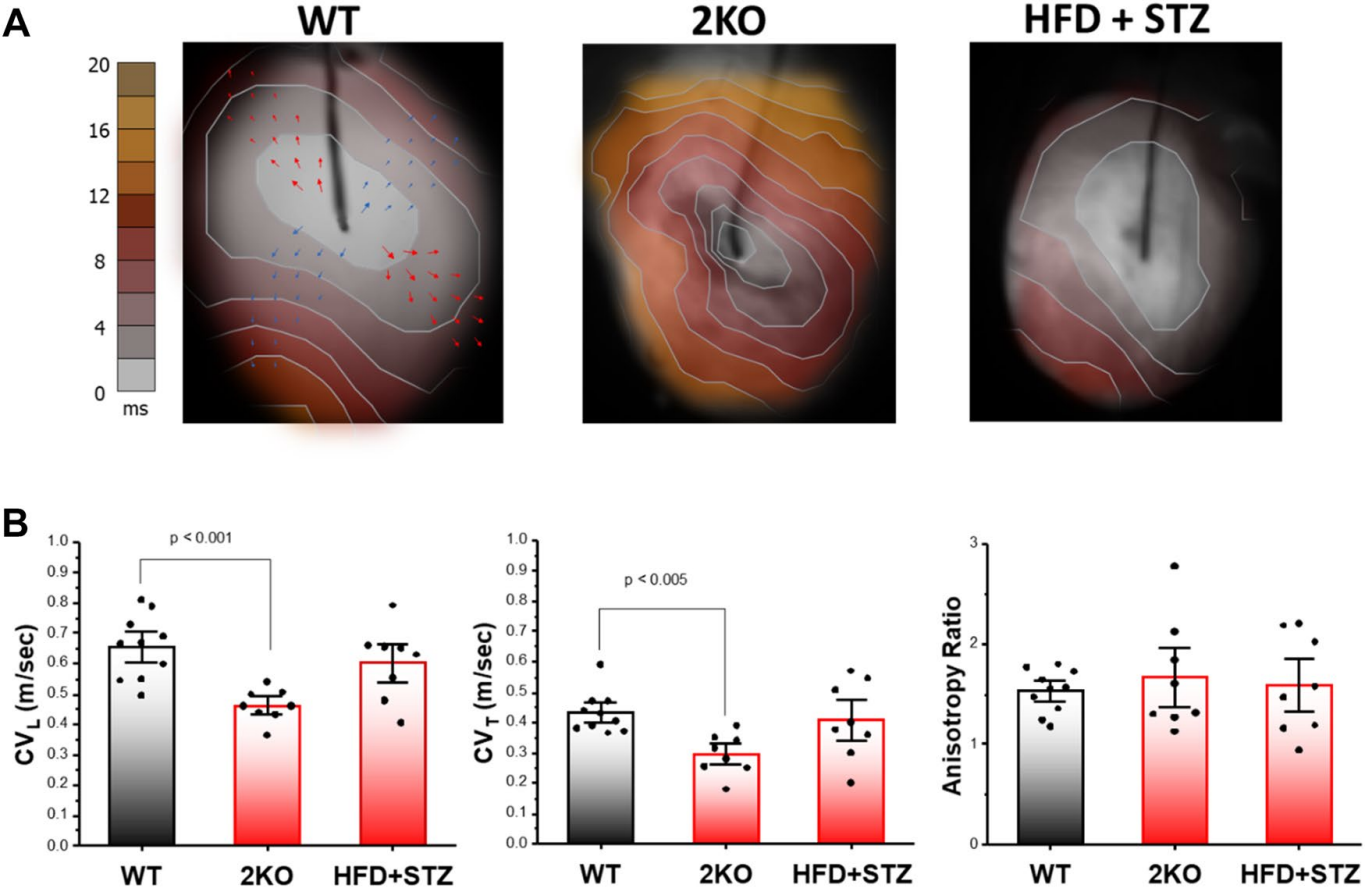
Optical mapping of action potentials from the anterior epicardial surface of Langendorff perfused hearts. **(A)** Activation maps showing impulse propagation in wild type (WT), *Lepr*^*db/db*^* eNOS*^*−/−*^ (2KO) and high fat diet plus low dose STZ-treated (HFD + STZ) mice. Stimulation was performed from the center of the mapping field of view at a cycle length of 110 ms. Arrows shown in WT example depict local conduction velocity vectors that were measured in the fast (longitudinal) and slow (transverse) direction of propagation. **(B)** Summary data for conduction velocity in the longitudinal (CV_L_) and transverse (CV_T_) directions, as well as CV_L_/CV_T_ (anisotropy ratio) in WT (n = 10), 2KO (n = 8) and HFD + STZ treated mice (n = 8). Data are mean ± SEM. P values were generated by Fisher’s least significant difference test for multiple comparisons.

**Figure 4:**
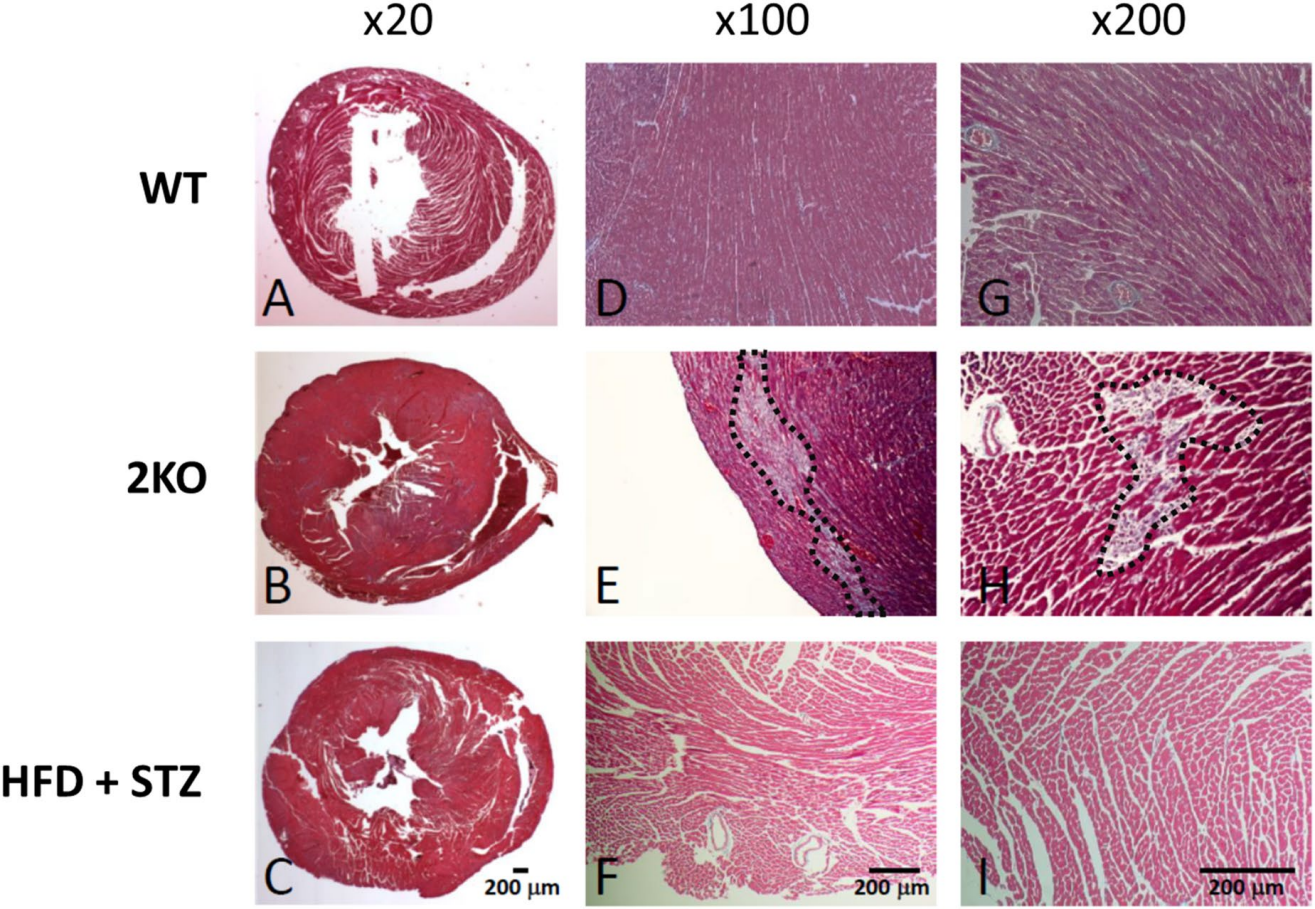
Histology (Masson’s trichrome stain) in wild type (WT), *Lepr*^*db/db*^* eNOS*^*−/−*^ (2KO) and high fat diet plus low dose STZ-treated (HFD + STZ) mice. Low magnification view from all three models **(A–C)** indicate the absence of large areas of scar typically associated with myocardial infarction. Diffuse fibrosis (area within black dashed line) was only observed in 2KO mice **(E,H)**. Each image is from a different tissue sample.

To determine the possible causes of arrhythmia (i.e., arrhythmia substrates), optical mapping of action potential activity was performed in Langendorff perfused hearts. Analysis of action potential impulse propagation in the ex vivo intact heart of 2KO mice revealed activation maps with slow impulse conduction (Fig. 3A, middle) indicated by the crowding of isochrone lines compared to wild-type controls (Fig. 3A. left). In contrast, activation maps in HFD + STZ mice were not different compared to wild-type controls (Fig. 3A, right). As shown for wild-type, local conduction velocity vectors were calculated in the direction of fast (longitudinal, red arrows) and slow (transverse, blue arrows) impulse propagation. Summary data (Fig. 3B) reveal significant slowing of conduction velocity in the longitudinal (left) and transverse (middle) directions for 2KO mice compared to wild-type; whereas HFD + STZ mice showed no difference in either direction compared to wild-type. The ratio of longitudinal to transverse conduction velocity (anisotropy ratio, Fig. 3B right panel) was not different across groups. Abnormal impulse conduction is commonly associated with myocardial fibrosis or scarring. Shown in Fig. 4 is representative histology for each mouse model. In the low magnification view (20X), neither model exhibited signs of significant scarring nor thinning of the LV chamber, which is typically associated with myocardial infarction. However, 2KO mice demonstrated significant diffuse myocardial fibrosis at six months, which was not observed in age-matched wild-type or HFD + STZ mice (Fig. 4), and might explain the slow conduction observed in hearts from 2KO mice.

**Figure 5:**
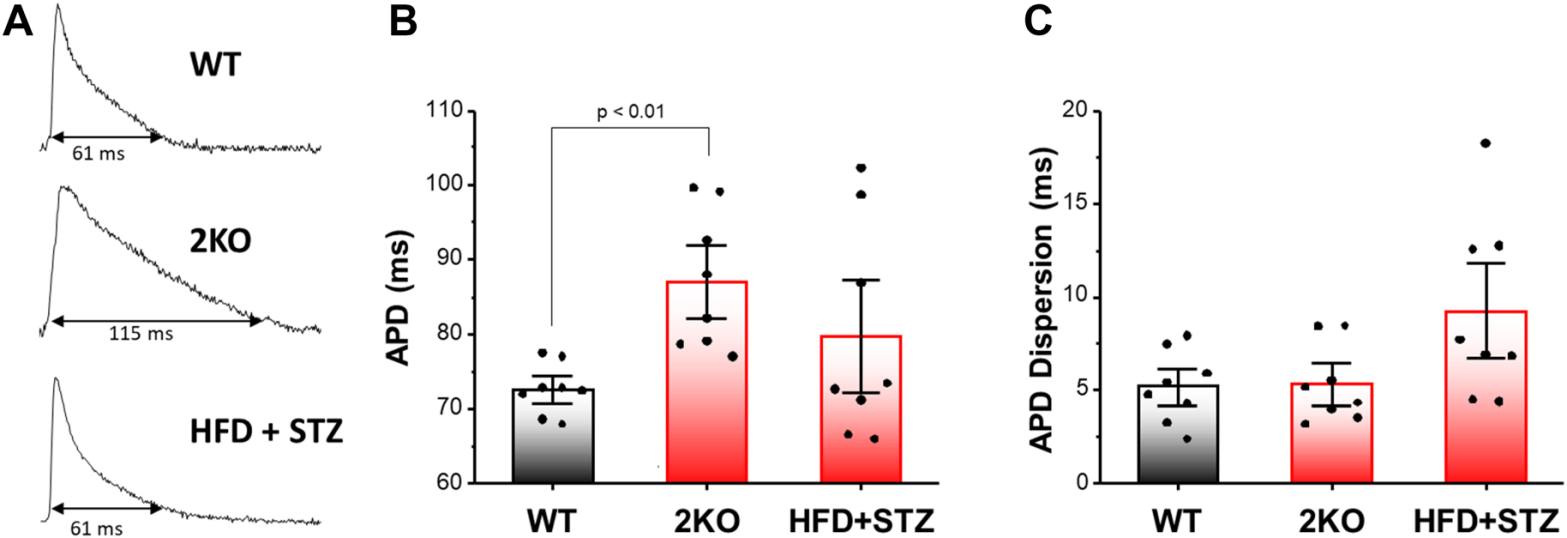
Action potential duration (APD) and APD dispersion in wild type (WT), *Lepr*^*db/db*^* eNOS*^*−/−*^ (2KO) and high fat diet plus low dose STZ-treated (HFD + STZ) mice. **(A)** Action potential traces show APD at 90% repolarization. Summary data for APD **(B)** and APD dispersion **(C)**. Data are mean ± SEM. P values were generated by Fisher’s least significant difference test for multiple comparisons.

We next examined abnormal action potential repolarization as another important arrhythmia substrate. Action potential traces recorded during steady state pacing (110 ms cycle length) suggest repolarization is significantly delayed in 2KO compared to wild-type and HFD + STZ mice (Fig. 5, Panel A). In summary data, APD was significantly prolonged in 2KO mice, but not in HFD + STZ, compared to wild-type mice (Fig. 5, Panel B). APD dispersion was determined as the standard deviation of APD measured from the RV base, RV apex, septum, LV base, and LV apex (five locations) within each heart tested. No significant difference was observed between groups, but there was a trend toward increased APD dispersion in HFD + STZ mice (Fig. 5, Panel C) compared to wild-type. In sum, both slow CV and prolonged APD, which are well-described arrhythmia substrates, were only observed in 2KO mouse hearts.

**Figure 6:**
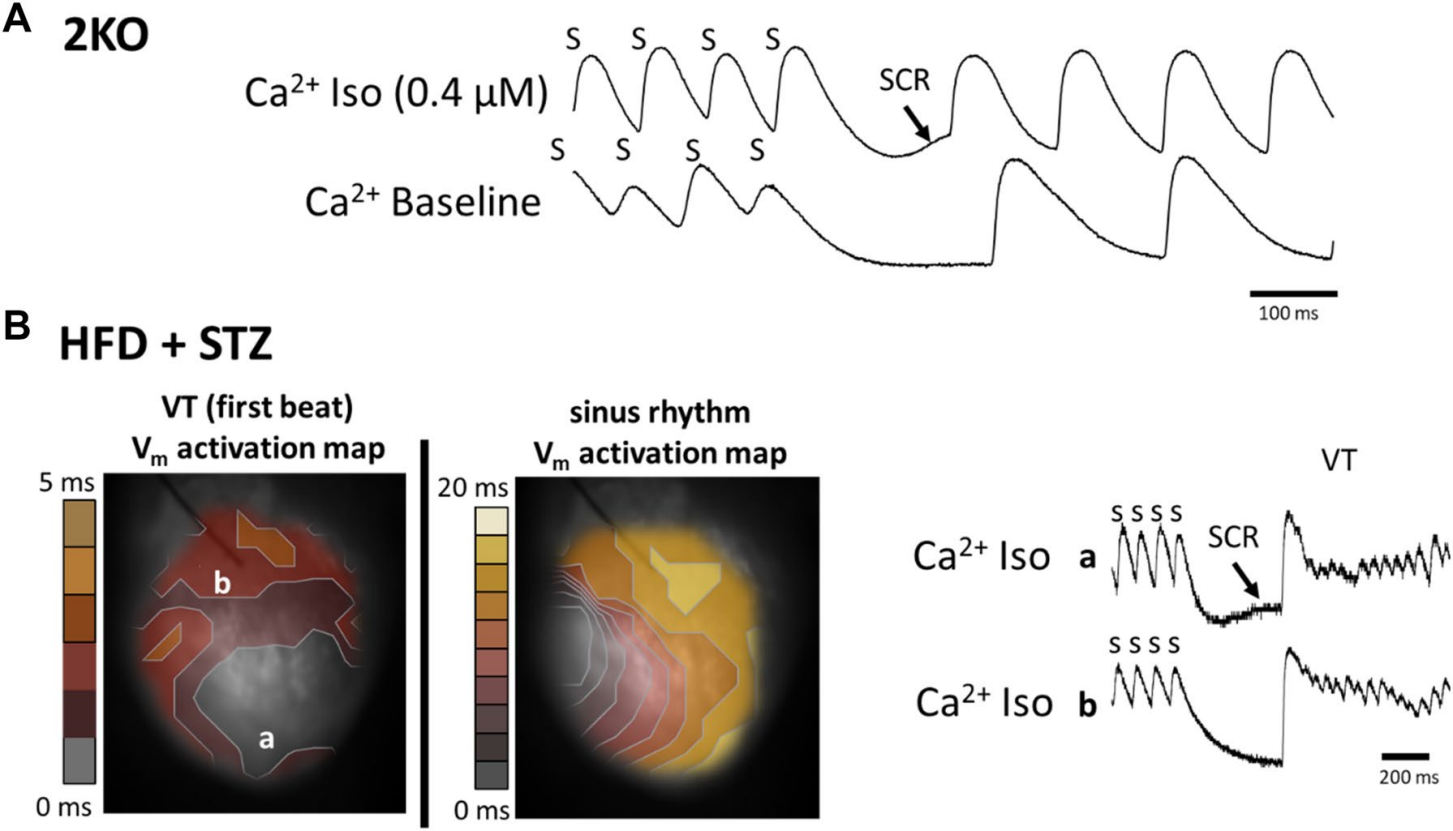
Examples of arrhythmias induced by isoproterenol (Iso) challenge and spontaneous calcium release (SCR) in *Lepr*^*db/db*^* eNOS*^*−/−*^ (2KO) and high fat diet plus low dose STZ-treated (HFD + STZ) mice. **(A)** Calcium transients with evidence of spontaneous calcium release (SCR, arrow) following the termination of rapid pacing (S) only in the presence of Iso (top trace). **(B)** Dual voltage-calcium optical mapping results. Activation maps were determined from action potentials measured simultaneously with calcium transients. The site of arrhythmia initiation (VT first beat, left panel **B**) is associated with the location of SCR (site a), rather than the absence of SCR (site b) following rapid pacing (s), as depicted in the calcium transient traces (right, Panel **B**). The activation map corresponding to sinus rhythm is shown in the right panel as a reference. Note the difference in time scale between the two maps.

In both mouse models, arrhythmias were more commonly observed with isoproterenol challenge, which we have previously shown is associated with spontaneous calcium release^37^. Calcium transient recordings from a 2KO mouse during isoproterenol revealed a typical pattern for spontaneous calcium release (Fig. 6A), as indicated by a slow rise in intracellular calcium during diastole immediately following termination of rapid pacing (SCR, arrow). In contrast, no spontaneous calcium release was observed from the same mouse/site under baseline conditions in the absence of isoproterenol. Similar results were observed in HFD + STZ-treated mice (Fig. 6B). Using dual Ca^2+^ and voltage optical mapping, the activation map for the first spontaneous beat of VT that was induced by rapid pacing (Fig. 6B, left map) shows the site of earliest activation (grey contour) corresponding to the site of maximum spontaneous calcium release (Panel B, site a, top trace). In contrast, at a site remote from earliest activation (site b, bottom trace), there is no evidence of spontaneous calcium release preceding VT.

These results are expected if the first beat of VT is triggered activity due to spontaneous calcium release. Additionally, the site of earliest activation during VT is different than the site of earliest activation during sinus rhythm (Fig. 6B, right map), indicating that the first spontaneous beat after rapid pacing is not a sinus beat. Across both 2KO and HFD + STZ mouse models, evidence of spontaneous calcium release was observed in three of five animals tested. Importantly, no spontaneous calcium release was observed in wild-type mice with or without isoproterenol. These data are consistent with the high incidence of arrhythmia during isoproterenol challenge (Table 1) and suggest spontaneous calcium release as the underlying cause in both DKD models.

Several plausible mechanisms of kidney-initiated arrhythmogenesis have been proposed, including neurohumoral abnormalities and altered concentrations of circulating factors. To address the latter possibility, we hypothesized that the small molecules trimethylamine N-oxide (TMAO) and asymmetric dimethylarginine (ADMA) promote arrhythmias in our models. TMAO and ADMA are putative uremic solutes derived from the gut microbiome and have been implicated in coronary artery disease pathogenesis^38,39^. As an initial screen, we assayed TMAO and ADMA levels in plasma from wild-type and 2KO mice. Table S2 (supplement) demonstrates that concentrations of both uremic solutes were significantly elevated in 2KO plasma, and at magnitudes that are similar to blood levels in humans with CKD^40–54^.

**Figure 7:**
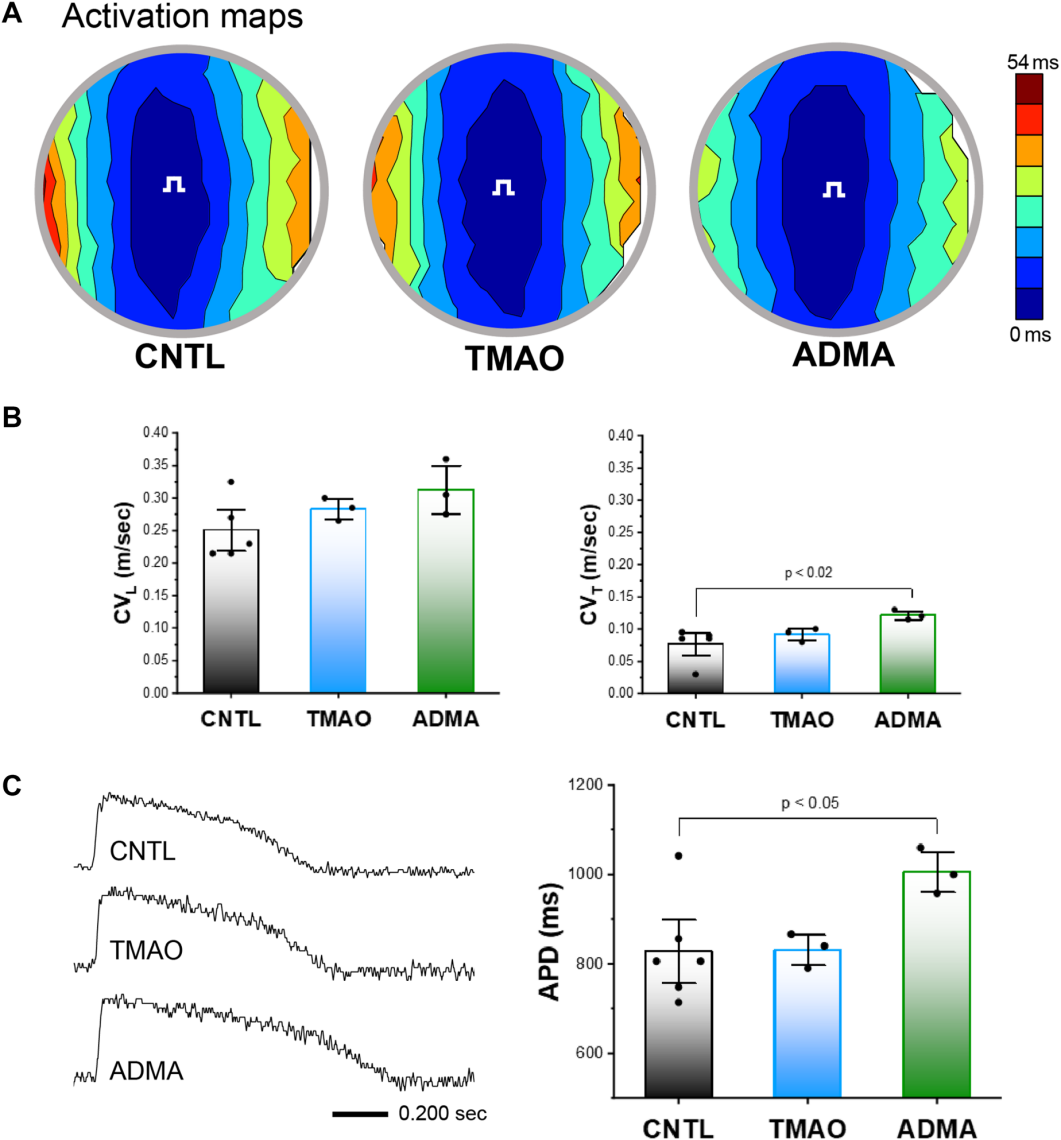
Effects of TMAO and ADMA on impulse conduction in human cardiac myocyte monolayers. **(A)** Activation maps showing impulse propagation in response to TMAO (100 µM), ADMA (10 µM) or DMSO vehicle only (control, CNTL). Stimulus symbol (square wave) is the site of point infrared stimulation. **(B)** Summary data for conduction velocity in the longitudinal (CV_L_) and transverse (CV_T_) directions. Effects of TMAO and ADMA on action potential duration (APD) in human cardiac myocyte monolayers **(C)**. Single site action potentials (left) in the presence of TMAO (100 µM), ADMA (10 µM) or DMSO vehicle only (control). Summary data (right) for APD. Data are mean ± SEM. P values were generated by Fisher’s least significant difference test for multiple comparisons.

To test whether TMAO and ADMA could precipitate arrhythmias, we exposed human ventricular cardiac myocyte monolayers to concentrations that simulate circulating concentrations and used optical mapping techniques to measure action potentials during steady state infrared point stimulation, as described previously^55^. Examples of activation maps show no qualitative differences in impulse propagation between TMAO and ADMA compared to controls (Fig. 7A, contour maps). Summary data (Fig. 7B) show that ADMA slightly increased conduction velocity only in the transverse direction. However, the physiological difference is small, and super-normal conduction velocity is not an arrhythmia substrate, and thus unlikely to explain our arrhythmia results observed ex vivo. In contrast, action potential traces suggest that APD is prolonged with ADMA compared to control (Fig. 7, Panel C, left). Summary data (Fig. 7, Panel C, right) demonstrate that ADMA significantly increased APD, which is consistent with 2KO hearts ex vivo. APD differences with TMAO were not significantly different compared to control.

## Discussion

SCD due to VT is a major cause of mortality in CKD and ESRD, and the electrophysiologic mechanisms may be unique in these patient populations. Animal models that recapitulate the pathophysiology of CKD-associated VT are lacking^56^. In this report, we demonstrate premature mortality and VT inducibility in the majority of both genetic (*Lepr*^*db/db*^* eNOS*^*−/−*^) and inducible (HFD plus low dose STZ) mouse models of DKD. In both models, arrhythmia inducibility was more common with isoproterenol than programmed electrical stimulation and characterized by triggered activity due to spontaneous calcium release. The 2KO mice also demonstrated delayed conduction velocity and prolonged APD. Plasma concentrations of two uremic solutes, which have been implicated in CVD pathophysiology, ADMA and TMAO, were significantly higher in 2KO compared to wild-type control mice. Incubation of human cardiac myocytes with ADMA, at concentrations that approximated blood levels of ESRD patients on dialysis, also resulted in prolonged APD, as determined by optical mapping methods.

Mice with the combined *Lepr*^*db/db*^* eNOS*^*−/−*^ mutant genotypes have not previously been examined for arrhythmia inducibility. This is important because the individual *Lepr*^*db/db*^ and eNOS^−/−^ mutations yield some features of DKD, such as glomerulosclerosis and albuminuria, but the combined 2KO mutation represents a more reliable phenocopy for DKD^14^. In particular, 2KO is required to cause decreased GFR^14,19^, which has been the kidney phenotype most reliably associated with heart disease^2^, and the source of uremic toxins, due to inadequate clearance by the kidneys^57^. Several previous studies have separately examined arrhythmia substrates in *Lepr*^*db/db*^^20,21^ and *eNOS*^*−/−*^^22,24,25^ mice. Our data confirm many prior findings with these mice, particularly increased APD, delayed after-depolarizations and abnormal calcium handling^20,24,25^ which is consistent with prolonged QTc in CKD patients^58^.

Because the arrhythmias could be ramifications of *Lepr* mutation or *eNOS* gene deletion per se, rather than mimicking DKD, we also conducted experiments in a dissimilar, inducible model of DKD. Mice fed a HFD for six months, which were then treated with low dose STZ at three months, also developed obesity, hyperglycemia, and insulin resistance, reminiscent of type 2 diabetes, as well renal dysfunction characterized by glomerulosclerosis, albuminuria and decreased GFR^19^. Similar to results observed in the 2KO mice, the HFD + STZ mice demonstrated arrhythmia inducibility with isoproterenol and PES at a similar frequency, triggered activity, and spontaneous calcium release. Taken together, we conclude that the consistent arrhythmia phenotype between the two models indicates that DKD is causally related to arrhythmia propensity.

Even though total arrhythmia incidence and prevalence of isoproterenol-induced arrhythmias were similar for both models (Table 1), they exhibited some differences in optical action potential results. For example, CV was decreased and APD was increased in 2KO mice, whereas these were unchanged in HFD + STZ mice. Decreased CV and prolonged APD are arrhythmia substrates; however, when both are present, they cancel and have no effect on cardiac wavelength (= APD × CV), which is an important determinant of reentrant excitation induced by PES. Therefore, this could explain why PES-induced arrhythmias for 2KO mice was relatively uncommon and of similar incidence compared to HFD + STZ mice, even in the presence of decreased CV and prolonged APD. While the underlying molecular causes of the arrhythmia substrates are beyond the scope of this study, the unchanged anisotropic ratio for all groups (Fig. 3B) suggests that the slowing of conduction in 2KO mice was not due to changes in connexin protein expression^59^. Crucially, both 2KO and HFD + STZ models of DKD exhibited a similarly high level of isoproterenol-induced arrhythmias that was associated with spontaneous calcium release (Fig. 6). These results are consistent with previous reports of arrhythmia mechanisms in a rat model of CKD^60^, and suggest that abnormal impulse formation mediated by calcium dysregulation is an arrhythmia substrate that is common to DKD. Importantly, *Lepr*^*db/db*^* or eNOS*^*−/−*^ alone do not replicate the optical action potential and arrhythmia phenotypes we observed in 2KO mice (combined *Lepr*^*db/db*^* eNOS*^−/−^). Previous studies have shown that *Lepr*^*db/db*^ mice exhibit slow conduction^61^, but evidence for spontaneous calcium release is conflicting^62,63^. Similarly, eNOS deficiency has been associated with fibrosis^64^, which may explain conduction slowing that we observed. However, arrhythmia due to spontaneous calcium release in *eNOS*^−/−^ mice is not established.

In both the 2KO and HFD + STZ mice we observed a preponderance of isoproterenol-induced arrhythmias that are mediated by abnormal impulse formation, which are typically resistant to ICD treatment. These results are consistent with limited data in clinical trials that included CKD or ESRD patients. In a large study of Medicare beneficiaries with ICDs for primary arrhythmia prevention, the greatest risk factor for subsequent death was CKD^65^. Subgroup analysis of MADIT-II demonstrated no benefit of ICDs in patients with CKD and EF < 35%^8^. Although there was benefit in the EF > 35% group^8^, a recent prospective trial demonstrated no ICD benefit in subjects with ESRD and EF > 35%^7^. ESRD patients with ICDs are more likely to receive appropriate shocks, but have a higher death rate, compared to non-ESRD groups^57,66–69^.

To test the possibility that circulating factors associated with DKD could induce arrhythmia substrates, we focused on two uremic solutes (ADMA and TMAO) that have been implicated in CVD pathophysiology. Although TMAO has been more widely examined as a candidate for uremic cardiomyopathy, we recently found in a large observational diabetic cohort study, that ADMA, but not TMAO, was associated with all-cause and cardiovascular mortality (Sapa H et al., manuscript under review). These data are consistent with our in vitro findings that ADMA, but not TMAO, was associated with prolonged APD. Moreover, our in vitro findings are consistent with high levels of ADMA in plasma and prolongation of APD that we observed in ex vivo 2KO hearts. One plausible reason for similarities between the ex vivo and in vitro findings may be that the mouse model contains global *eNOS* deletion, and the mechanism of ADMA toxicity is at least partly due to nitric oxide inhibition^70^. More investigation will be required before concluding that ADMA is a major culprit in the pathogenesis of CKD-associated SCD, particularly since it is merely one of dozens of uremic toxins that have been identified to date^71,72^ and there are likely other uremic toxins that can induce arrhythmia substrates^73^. Nevertheless, to our knowledge the present study is the first to employ in vitro optical mapping to characterize arrhythmia substrates in human cardiac myocytes exposed to uremic toxins. One clear application of this method is the potential for high throughput screening, both for combinations of uremic toxins as instigators of arrhythmias, as well as for testing antiarrhythmic drug efficacy under conditions that simulate CKD or DKD.

Herein we describe two mouse models that faithfully phenocopy DKD pathophysiology, with associated premature mortality and increased risk for arrhythmia, similar to humans with DKD. Importantly, despite one model being genetic and the other inducible, the arrhythmia propensity is strikingly similar, suggesting that DKD is causally related to arrhythmogenesis. Moreover, our findings with novel in vitro optical mapping techniques demonstrated that uremic toxins replicate, in part, the arrhythmia substrates, suggesting a mechanism for DKD-induced arrhythmias and SCD. Interestingly, the propensity for arrhythmias due to abnormal impulse formation in both DKD models is consistent with observations in humans that ICDs are less effective in patients with CKD. In sum, the present study elucidates mechanisms of cardiovascular disease mortality associated with DKD. Finally, we propose that our findings and screening strategies could lead to more effective future treatments in the vulnerable DKD patient population.

## Methods

### Mouse DKD models

BKS.Cg-Leprdb Nos3tm1Unc/RhrsJ (*Lepr*^*db/db*^* eNOS*^*–/–*^) mice were purchased from Jackson Laboratory (Bar Harbor, ME). *Lepr*^*db/*+^ *eNOS*^+*/–*^ on C57BLKS/J genetic backgrounds were intercrossed to generate experimental groups. *Lepr*^*db/db*^* eNOS*^*–/–*^ mice were genotyped by PCR and develop DKD, as previously described^19^. Methods to induce type 2 diabetes and DKD were described previously^19^. Briefly, wild-type male C57BLKS/J mice were fed a high fat diet (Teklad TD.06414, 60.3% fat, 21.3% carbohydrate, 18.4% protein; Harlan Laboratories, Indianapolis, IN) for six months. At three months, mice were administered low dose streptozotocin (STZ, 65 μg/g i.p. daily for two consecutive days). HFD + STZ mice experienced GFR declines at ages similar to *Lepr*^*db/db*^* eNOS*^*–/–*^ mice^19^. Fasting blood glucose was assayed weekly by glucometer, and diabetes was defined by concentrations consistently greater than 200 mg/dL.

### Optical mapping in Langendorff perfused mouse heart

Mice were injected with heparin (500 units per mouse) and then anesthetized i.p. with a ketamine (100 mg/Kg)/xylazine (10 mg/kg) mixture. Hearts were excised and cannulated for Langendorff perfusion with 34°–36° O_2_-bubbled Tyrode solution containing (mM) NaCl 140, KCl 4.5, CaCl_2_ 1.25, MgCl_2_ 0.7, HEPES 10, and dextrose 5.5 (pH 7.4) and placed in a heated custom-built Plexiglas chamber, as described previously^74^. Electrocardiograms, perfusion pressure (40–60 mmHg), and bath temperature were measured continuously during the entire experiment. Blebbistatin (6 μM) was included in the perfusate to eliminate motion artifact during optical recordings. For optically mapping action potential activity, hearts were perfused with a bolus of the voltage-sensitive dye di-4-ANEPPS (30 μM). Optical action potentials were recorded simultaneously from the mouse anterior RV/LV surface within a 10 × 10 mm mapping field of view. Fluorescence was excited with uniform light from a high-power green LED filtered at 515 ± 5 nm, then collected (custom, high magnification tandem lens assembly) and transmitted (emission filter > 610 nm) to a CMOS based camera (MiCAM ULTIMA, SciMedia, Costa Mesa, CA). Frames (100 × 100 pixels) were acquired at a rate of 2000 Hz and binned in software (5 × 5) yielding an equally spaced 20 × 20 array of action potential recordings with 0.5 mm inter-pixel spatial resolution. Other than pixel binning, no additional spatial or temporal filtering was utilized. For dual voltage-Ca^2+^ optical mapping, previously described techniques were utilized^35^.

In every mouse heart, steady state ventricular epicardial pacing was performed at cycle lengths ranging from 160 to 60 ms with 10 ms decrements. Then, arrhythmia inducibility was determined by programmed electrical stimulation with up to three premature stimuli (S1-S4 pacing protocol) and with an isoproterenol challenge (400 nM). Optical mapping studies were all conducted by a blinded investigator, and no animals were excluded.

### Optical mapping in human ventricular myocyte monolayers

Human cardiac myocytes derived from induced pluripotent stem cells were purchased from Cellular Dynamics Inc (Madison, WI). Cells (6.6 × 10^4^ per well) were plated on fibronectin-coated, 3D-aligned nanofiber 96-well plates (Nanofiber Solutions, Dublin, OH) to simulate adult (i.e. anisotropic) structure and impulse propagation. Culture media was changed every 2 days, until day 14–20 when experiments were performed.

Fluorescence recordings of action potentials were performed using a custom-designed inverted macroscope with ports for optical pacing with infrared laser light and fluorescence excitation with a high-power LED as described previously^55^. Fluorescence from a single well (535 ± 30 nm) was focused onto a MiCam02-HR CCD camera (SciMedia) with a 6 mm × 7.5 mm field of view. The CCD was configured for 10 × 10 pixel binning with additional 2 × 2 binning in software, resulting in 20 × 14 binned pixels. No subsequent spatial or temporal filtering was utilized. Monolayers were incubated with Tyrode’s solution (140 NaCl, 4.5 KCl, 0.73 MgCl_2_, 10 HEPES, 5.0 dextrose, 1.25 CaCl_2_) containing 1/2 × FluoVolt (Sigma-Aldrich, St. Louis, MO) for 15 min. After incubation, all monolayers were then washed with normal Tyrode’s solution before recordings were performed at room temperature.

Cells were paced with infrared laser light at a cycle length of 0.5 Hz, during which action potentials were measured. A 1475 nm diode laser (MCM-102, Seminex, Peabody, MA) was used for pacing hCM monolayers. The laser was coupled into a 600 μm multi-mode optical fiber (Ocean Optics, Dunedin, FL) and attached to the optical port. Recordings were performed in wells with Tyrode solution containing trimethylamine N-oxide (TMAO, 10–100 μM), asymmetric dimethyl arginine (ADMA, 1–10 μM) or DMSO vehicle only. No electro-mechanical uncouplers were used in any of these experiments. Samples were tested in random order.

### Histology

Mouse hearts were removed, dissected into three coronal sections and immediately fixed in 4% paraformaldehyde. Fixed tissue was embedded in paraffin blocks, and 10 μm microtome sections were obtained from the middle coronal section, which contained both ventricles. De-paraffinized sections were then stained with Masson’s trichrome, according to guidelines from MilliporeSigma (HT15), as previously described^75^. Images at magnifications ranging from 20 to 200× were obtained using a Nikon Eclipse E600 microscope (Tokyo, Japan) and a Spot RT3 digital camera (Sterling Heights, MI).

### Liquid chromatography–mass spectrometry (LC–MS/MS) analysis

Mouse plasma (5 µl volume) was injected into a Luna Silica column (250 × 4.6 mm 5 μm silica 000G-4274-E0, 150 × 2 mm, 5 µm silica, 00F-4274-B0, Phenomenex, Torrance, CA). LC–MS/MS analysis employed a Shimadzu Prominence LC system coupled to an API 4000 Q-TRAP mass spectrometer (AB Sciex, Framingham, MA). Binary flow was generated to resolve the analytes by using mobile phases (0.1% propionic acid in H_2_O) and (0.1% acetic acid in methanol) at 0.2 ml/min flow rate. The analytes, TMAO, TMAO-d9, ADMA and ADMA-d7 were monitored using electrospray ionization in positive-ion mode with multiple reaction monitoring of precursor and characteristic product ion transitions. Calibration curves were generated, to which internal standards (TMAO-d9 and ADMA-d7) were added and analyzed by linear regression using Analyst software (version 1.6, Framingham, MA) for TMAO and MultiQuant SignalFinder software (version 3.0, Framingham, MA) for ADMA quantification.

### Data analysis

Data analysis including determination of action potential duration (APD) and local conduction velocity was performed for all steady state pacing cycle lengths tested using custom software designed for analysis of optically recorded action potentials^36,55,76^. Briefly, activation times were assigned for each action potential by identifying the greatest positive change in fluorescence during the action potential upstroke. Local conduction velocity at each recording site was calculated during steady-state pacing, from which longitudinal and transverse propagation velocity (if apparent) was determined by averaging corresponding sites. VT/VF was defined as lasting > 3 beats. After optical mapping, hearts were prepared for histological analysis.

### Statistics

All experiments were conducted a minimum of three times. Chi square test was used to compare Kaplan–Meier survival curves. Fisher’s exact test was used to compare arrhythmia incidence between the wild-type and experimental DKD groups. Ex vivo and in vitro data from three groups were analyzed by one-way ANOVA and Fisher’s least significant difference test for multiple comparisons (Origin 2019b, Northampton, MA). Results are expressed as mean ± standard error and a P value < 0.05 was considered statistically significant.

### Study approval

All protocols and procedures were approved by the Institutional Animal Care and Use Committee of Case Western Reserve University and conducted in accordance with the Guide for the Care and Use of Laboratory Animals (National Academies Press, 2011). This study complies with the Essential 10 ARRIVE criteria.

## Supplementary Information

Supplementary Video S1.

Supplementary Video S2.

Supplementary Information.
