# Supplementary material for: Ventricular arrhythmias in mouse models of diabetic kidney disease
Source: Sci Rep. 2021 Oct 18;11:20570. doi: 10.1038/s41598-021-99891-9 (PMC8523538; doi:10.1038/s41598-021-99891-9)
Supplement: Supplementary file 3 — Supplementary Information. [file 41598_2021_99891_MOESM3_ESM.pdf]

Ventricular Arrhythmias in Mouse Models of Diabetic Kidney Disease

Kenneth R. Laurita[1,2], Shenaz Khan[1], Tracy McMahon[1], Adrienne T. Dennis[1], Vincent Li[1], Robert Gaivin[1], Hima Sapa[4], Ji-dong Fu[1,3], Jeffrey R. Schelling[1,5]

1. Department of Medicine, Case Western Reserve University, MetroHealth campus, Cleveland, OH 44109
2. Department of Biomedical Engineering, Case Western Reserve University, Cleveland, OH 44106
3. Current address: Department of Physiology and Cell Biology, The Ohio State University, Columbus, OH 43210
4. Department of Medicine, Case Western Reserve University, University Hospitals Cleveland, Cleveland, OH 44106
5. Department of Physiology and Biophysics, Case Western Reserve University, Cleveland, OH 44106

Supplemental Information

Supplemental Tables

Mouse strain	Wild-type	2KO	HFD + STZ
N	12	10	24
Maximum body wt (g)	25.8 ± 1.4*	44.3 ± 1.2	41.6 ± 1.4
Plasma glucose (mg/dl)	116.2 ± 6.6*	579.8 ± 20.2	414.1 ± 22.8

Table S1. Characteristics of the three experimental groups, wild-type, Lepr$^{db/db}$ eNOS$^{-/-}$ (2KO) and high fat diet plus low dose streptozotocin (HFD + STZ)-treated C57Bl-KS/J mice. Data are mean ± SEM. *P <0.01 compared to other groups by Student's t-test.

	TMAO (µM)	ADMA (µM)
**Normal human population**	2.0-3.4 (42-44, 51)	0.40-0.77 (45-47, 54)
**Human CKD**	7.9-24.1 (40, 42-44)	0.46-1.04 (41, 45-50)
**Humans on dialysis (ESRD)**	77-102 (43, 44, 51-53)	0.82-1.06 (46, 49, 52, 54)
**Wild-type**	7.62 ± 1.61	0.87 ± 0.10
**2KO**	53.44 ± 22.17*	1.69 ± 0.29**
**HFD + STZ**	6.20 ± 2.71	0.89 ± 0.10

Table S2. Concentrations of TMAO and ADMA in humans with and without kidney disease (upper three rows). Data from humans are ranges of mean values from individual studies. Reference numbers are in parentheses. Plasma values from wild-type (N=6), Lepr$^{db/db}$eNOS$^{-/-}$ (2KO, N=8) mice, and high fat diet plus low dose streptozotocin (HFD + STZ)-treated C57Bl-KS/J mice (N=4) are shown in lower three rows. Data

from animal studies are mean ± SEM. *P <0.01 compared to other two groups by one-way ANOVA. **P <0.05 compared to other two groups by one-way ANOVA.

Supplemental Videos (Legends)

S1 Video. Shown is an example of normal sinus rhythm in a wild-type mouse. Raw fluorescence is color coded in shades of red where black, corresponding to resting membrane potential, is transparent and brightest red corresponds to maximum membrane depolarization. Raw fluorescence is superimposed on an image of the heart, where the pacing electrode is shown (black line) but not being utilized in this example. Each frame corresponds to 1 ms and the frame rate is not real-time (see time counter in upper right-hand corner).

S2 Video. Shown is an example of spontaneous ventricular tachycardia in a 2KO mouse. Raw fluorescence is color coded in shades of red where black, corresponding to resting membrane potential, is transparent and brightest red corresponds to maximum membrane depolarization. Raw fluorescence is superimposed on an image of the heart, where the pacing electrode is shown (black line) but not being utilized in this example. Each frame corresponds to 1 ms and the frame rate is not real-time (see time counter in upper right-hand corner).
